# Supplementary material for: The discovery of novel noncoding RNAs in 50 bacterial genomes
Source: Nucleic Acids Res. 2024 Apr 22;52(9):5152–65. doi: 10.1093/nar/gkae248 (PMC11109978; doi:10.1093/nar/gkae248)
Supplement: gkae248_Supplemental_Files [file gkae248_supplemental_files.zip › Narunsky 50 Genomes RNA Motifs Files.zip!/Publication/56-D_turgidum/Figures/LRC-56-22-NC_011661_1_IGR_69_783003_783274.sto.pdf]

LRC-56-22-NC_011661_1_IGR_69_783003_783274.sto.r2r

5′-○○○UUYUYR●AAA●●A●●●●●●RR●RYCUC●GRAYR●●R●CCUCO○YUG●UACUCAUAYA●●UU●YUCUCUCCCUCCCUUCYUUUY●●●●●UUUYUUURUUAUARY●CURURRR●●●U●RGY●UCA●○●●●AR●U●RARO○A●AY●A●A●UA●AAY○○○○○○○○○○○YR○RRA●A●●RR○○○○○○○○○○○○○○○
